# A Method for Screening Climate Change-Sensitive Infectious Diseases

**DOI:** 10.3390/ijerph120100767

**Published:** 2015-01-14

**Authors:** Yunjing Wang, Yuhan Rao, Xiaoxu Wu, Hainan Zhao, Jin Chen

**Affiliations:** 1 State Key Laboratory of Earth Surface Processes and Resource Ecology, College of Global Change and Earth System Science, Beijing Normal University, Beijing 100875, China; E-Mails: yunjingw@gmail.com (Y.W.); yuhanrao@mail.bnu.edu.cn (Y.R.) ; wuxx@bnu.edu.cn (X.W.); 2 School of Mathematical Sciences, Beijing Normal University, Beijing 100875, China; E-Mail: magnolia_hn@163.com

**Keywords:** climate change, infectious diseases, climate change-sensitive diseases, relative sensitivity, screening method

## Abstract

Climate change is a significant and emerging threat to human health, especially where infectious diseases are involved. Because of the complex interactions between climate variables and infectious disease components (*i.e.*, pathogen, host and transmission environment), systematically and quantitatively screening for infectious diseases that are sensitive to climate change is still a challenge. To address this challenge, we propose a new statistical indicator, Relative Sensitivity, to identify the difference between the sensitivity of the infectious disease to climate variables for two different climate statuses (*i.e.*, historical climate and present climate) in non-exposure and exposure groups. The case study in Anhui Province, China has demonstrated the effectiveness of this Relative Sensitivity indicator. The application results indicate significant sensitivity of many epidemic infectious diseases to climate change in the form of changing climatic variables, such as temperature, precipitation and absolute humidity. As novel evidence, this research shows that absolute humidity has a critical influence on many observed infectious diseases in Anhui Province, including dysentery, hand, foot and mouth disease, hepatitis A, hemorrhagic fever, typhoid fever, malaria, meningitis, influenza and schistosomiasis. Moreover, some infectious diseases are more sensitive to climate change in rural areas than in urban areas. This insight provides guidance for future health inputs that consider spatial variability in response to climate change.

## 1. Introduction

According to the Intergovernmental Panel on Climate Change’s (IPCC) fifth report, the Earth system is experiencing an unequivocal warming, and will likely become dangerously warmer during this century [1]. In addition to climate change’s impacts on the physical Earth system, climate change also substantially affects human health, especially where infectious diseases are involved [2,3]. There are three essential components for most infectious diseases: an agent (or pathogen), a host (or vector) and a transmission environment [4]. For most climate-sensitive infectious diseases, climate change may impact the whole process of a disease’s development [5,6], including the survival, reproduction, or distribution of disease pathogens and their hosts, as well as the availability and means of such pathogens’ transmission environments. For example, temperature increases impact the spread of malaria; Leeson [7] and Patz *et al*. [8] revealed the influence of increasing temperature on the pathogen, while Beck-Johnson *et al.* [9] described the relationship between temperature and the population dynamics of the host (*i.e.*, *Anopheles* mosquitos). Considerable attention has been focused on the influence of climate change on infectious diseases [10,11]. However, previous studies have mainly concentrated on identifying the impacts of climate change on some component of the development of infectious diseases. The sensitivity of infectious diseases to climate change remains unclear because of the complex interactions between climate variables and the three key components of infectious disease development. As a result, quantitative screening (from a systematic perspective) for the infectious diseases that are sensitive to climate change is still a challenge without adequate research.

The wealth of literature dealing with disease’ sensitivities to the climate mainly involve correlation or regression analysis. For example, Pearson’s correlation coefficients can be used to describe the crude relationship between average weather variables and logarithm of diarrhea morbidity [12]. A further study measured the link between climate change and diarrhea-associated morbidity and predicted the dynamics of diarrhea-associated morbidity in subtropical Taiwan by applying a climate variation-guided Poisson regression model [13]. However, such correlation analysis can only identify the static relationship between climate variables and infectious disease related parameters (*i.e.,* morbidity). In addition, other time series methods, such as cross-correlation analysis, provide opportunities for recognizing time lags and the temporal correlation between incidence data and climate data. They also assume that diseases respond linearly to climate variables, while research has revealed that climate change can have nonlinear influences on biological systems [14,15]. This indicates that the impacts on infectious diseases might be different under different climate regimes. Therefore, those methods that assume linear relationships would fail to detect nonlinear relationships. On the other hand, Woolf’s relative risk statistic (RR) [16], which has been applied to estimate the increased risk of contracting a disease before and after a certain condition (or trait) is present [17], has become a standard measure in biomedical research [18]. Later, many more statistical factors have been proposed, including haplotype relative risk (HRR) [17], which is used to calculate the risk of disease in the presence of particular antigens or phenotypes. Potential impact fraction (PIF) was also developed by the WHO, to estimate the disease burden caused by the change of a given risk factor based on relative risk measurement [19]. With RR as reference, if the relationship between infectious disease-related parameters and climate variables varies at two different climate statuses (*i.e.*, historical climate and present climate), it is possible to reasonably state that an infectious disease as sensitive to climate change through a metric that is similar to the RR, and employed to compare the risk variation corresponding to a specified change in a risk factor’s level.

Accordingly, this study proposes a statistically meaningful and comprehensive method to identify which infectious diseases are sensitive to climate change. Such a method is of particular importance for evaluating strategies to mitigate climate change’s influence on human health. This is especially the case when governments are making decisions concerning reasonable health care investments in the context of climate change. The rest of this paper is organized into four sections. Section 2 introduces the methodological development using the RR as a reference. Section 3 presents a case study in Anhui Province, China, to demonstrate the effectiveness of the proposed screening method. In this section the study area and data processing are also briefly introduced. The main content of Section 3 is a discussion concerning the screening results from climate change-sensitive diseases. The discussion compares the results with previous researches and illuminates the advantages and limitations of the screening method. In Section 4, a brief summary of the study is given.

## 2. Methodology

In epidemiology, the relative risk (RR) statistic was proposed to describe the increasing risk of contracting a disease in the presence of an exposure condition (*i.e.*, a drug/treatment or an environmental exposure) over that without such exposure condition. The general formula for calculating *RR* based on the exposure and non-exposure groups data (Table 1) is:

(1) $$RR=\frac{A/(A+B)}{C/(C+D)}$$

where *A* is the number of people contracting the disease in the exposure group, *B* is the number of people without the disease in the exposure group. *C* and *D* have identical definitions with *A* and *B* respectively but concern the non-exposure group. *RR* value equal to 1 means that there was no difference in risk between the two groups. *RR* value greater than 1 indicates an increased risk in the exposed group relative to the unexposed group, while *RR* valueless than 1 shows decreased risk in the exposed group [20].

**Figure 1:**
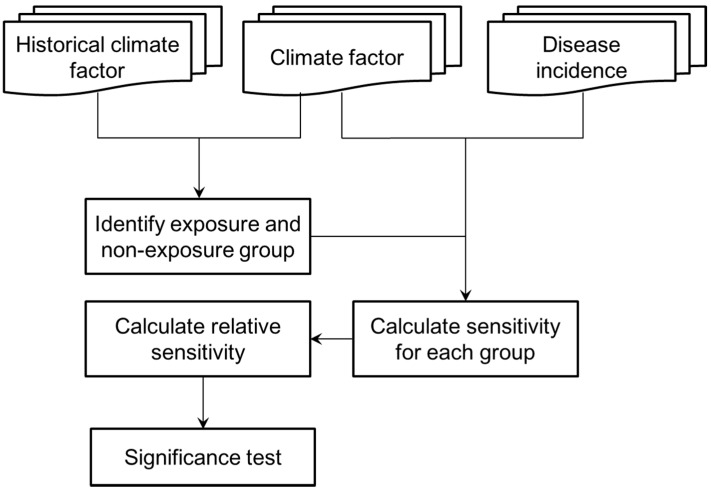
Flowchart of the calculation of Relative Sensitivity.

In order to screen the infectious diseases which are sensitive to climate change, we defined a new statistical indicator, Relative Sensitivity (RS), based on the relative risk statistic, to identify differences in the sensitivities of the infectious disease to climate variables at two different climate statuses (*i.e.*, historical climate and present climate). In detail, the group with the larger difference between two climate statuses is regarded as the exposure group, and the group with the smaller difference is defined as the non-exposure group. Thus, larger differences in the sensitivities of the two groups (*i.e.*, exposure and non-exposure groups) for an infectious disease indicate that the infectious disease is more sensitive to the change in the climate variables. The flowchart of the calculation of *RS* is shown in Figure 1, and a detailed introduction is given below. Here, it is noted that weather variables (*i.e.*, temperature and precipitation) have a direct influence on the epidemiology of infectious diseases, while climate change influences the epidemiology of infectious diseases through weather variables indirectly. In most literature, weather variables are also called climatic variables. Accordingly, we used climate variables or climatic variables instead of weather variables and have assumed that local weather variations can approximately reflect the average status of climate change.

**Table 1:** A 2×2 table for epidemiological relative risk calculation.

Group	Disease	Disease	Total
	Yes	No	
Exposure group	A	B	A+B
Non-Exposure group	C	D	C+D
Total	A+C	B+D	A+B+ C+D

**Figure 2:**
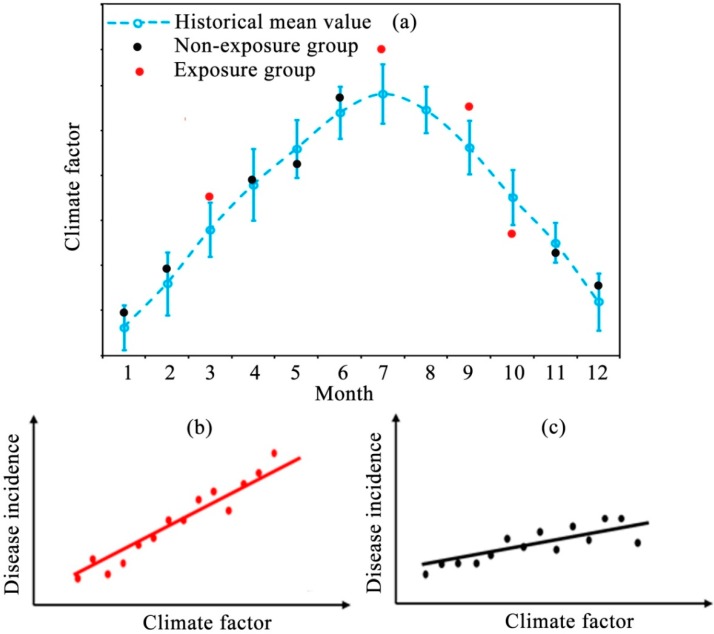
Schematic figure for calculating the Relative Sensitivity: (**a**) identification of the exposure and non-exposure groups; (**b**) linear model of the exposure group; (**c**) linear model of the non-exposure group.

The first step in the calculation of *RS* is to identify the exposure and non-exposure groups. In this study, the historical mean value (*HC_i_^mean^*) and the standard deviation (*HC_i_^std^*) of a climate variable (*C*) for the *i*th month (calculated from a 30 year historical dataset in this study) were used to define a threshold for dividing the exposure and non-exposure groups. For a study area, if the difference of the climate variable between the interested month (*PC_i_*) during the study period and the corresponding historical mean value (*HC_i_^mean^*) is larger than the standard deviation (*HC_i_^std^*) of the climate variable for the same month, the month is identified as an exposure month. Otherwise, the month will be identified as a non-exposure month (Figure 2a) (Equations (2) and (3)):

Exposure identification criteria:

(2) $$\left|PC_{i}-HC_{i}^{mean}\right|>HC_{i}^{std}$$

Non-exposure identification criteria:

(3) $$\left|PC_{i}-HC_{i}^{mean}\right|\leq HC_{i}^{std}$$

The second step is to obtain the sensitivity of a disease’s incidence to climate variables (e.g., temperature, humidity, precipitation, *etc.*) for each group (*i.e.*, the exposure and non-exposure groups). The sensitivity of a disease’s incidence to climate variables, hereinafter referred to as sensitivity, is defined as the slope of the linear model created by regressing disease incidence against corresponding climate variables. This slope reflects the change of disease incidence in response to a given change (usually one unit) in a climate variable:

(4) $$M_{1}:\;I=a_{1}C+b_{1}+\varepsilon$$

(5) $$M_{1}:\;I=a_{2}C+b_{2}+\varepsilon$$

where* M_1_* and *M_2_* indicate linear regression models of the exposure and non-exposure groups (Figure 2b,c), *I* stands for disease incidence, *C* stands for a climate variable, and *a_1_, a_2_* are the linear sensitivities of the exposure and non-exposure groups, respectively, which can be estimated using the least square method.

Finally the *RS* can easily be obtained using Equation (6), after a significance test. Since *a*_1_, *a*_2_ are slopes of different linear models, a hypothesis test can be carried out to examine whether these two linear models are statistically “parallel” (*i.e.*, *a*_1_ = *a*_2_). The null hypothesis for the test is that the sensitivity of the exposure group is statistically the same as the sensitivity of the non-exposure group (*i.e.*, *a*_1_ = *a*_2_), while the alternative hypothesis is that *a_1_* is significantly different from *a_2_* (*i.e.*, *a*_1_ ≠ *a*_2_):

(6) $$RS=\left|a_{1}-a_{2}\right|$$

(7) $$\begin{array}{cc} \begin{array}{cc} H_{0}: & a_{1}=a_{2} \end{array} & \begin{array}{cc} H_{1}: & a_{1}\neq a_{2} \end{array} \end{array}$$

Moreover, the hypothesis test is implemented using the *f* statistic [21], which is defined in Equation (8):

(8) $$f=\frac{\left(RSS_{H_{0}}-RSS_{H_{1}})/(n_{M}-1\right)}{RSS_{H_{1}}/(n_{D}-n_{M})})$$

*RSS_H_*_0_ and *RSS_H1_* are the residual sums of squares under the null hypothesis (*H*_0_) and the alternative hypothesis (*H*_1_) respectively, *n*_M_ is the number of models (*i.e.*, 2 in this research), while *n*_D_ is the number of data for all models. The *f* statistic denotes an *F-*distribution with the degree of freedom of *n*_M_−1 and *n*_D_−*n*_M_ (*i.e.*, *F*(*n*_M_−1, *n*_D_−*n*_M_)). Given a confidence level of 0.05 (*i.e.*, α = 0.05), the rejection criteria can be obtained as follows:

(9) $$f>F_{\alpha /2}(n_{M}-1,n_{D}-n_{M})$$

where *F_α_*_/2_(*n*_M_−1, *n*_D_−*n*_M_) is the α/2-quantile for the previously mentioned *F*-distribution. If the *f* statistic meets the rejection criteria, the *p*-value for the test is less than *α* (*i.e.*, *p* < 0.05 in this study), which means that the null hypothesis can be rejected (*i.e.*, *a*_1_ is significantly different from *a*_2_). Otherwise, we do not have enough evidence to reject the null hypothesis, which means that *a*_1_ is not significantly different from *a*_2_.

According to the procedures mentioned above, if the *RS* of an infectious disease can reject the null hypothesis, the disease can be regarded as a sensitive infectious disease relative to a change in a climate variable, and the larger the *RS* is, the more sensitive the infectious disease is to climate change.

As mentioned in the Introduction, climate change impacts all components in a disease’s development. The impacts may vary among different diseases and different stages in a disease’s development since they have different epidemiology. Temperature may play a crucial role in the development of some diseases, while other diseases are mainly affected by precipitation, wind or some other climate variables. Hence, it is difficult to investigate all these specific impacts (*i.e.*, impacts for different diseases, and for different stages in a disease’s development) using the same method. However, the objectives of this study are to investigate the general impacts of climate change on different diseases instead of specific impacts on different diseases, and then to find out the dominant climate variables, if any, for different diseases. In the interest of these objectives, it is reasonable to use the same univariate statistical methods to examine the general impacts of climate change on infectious diseases.

## 3. Application

### 3.1. Study Area and Data Processing

**Figure 3:**
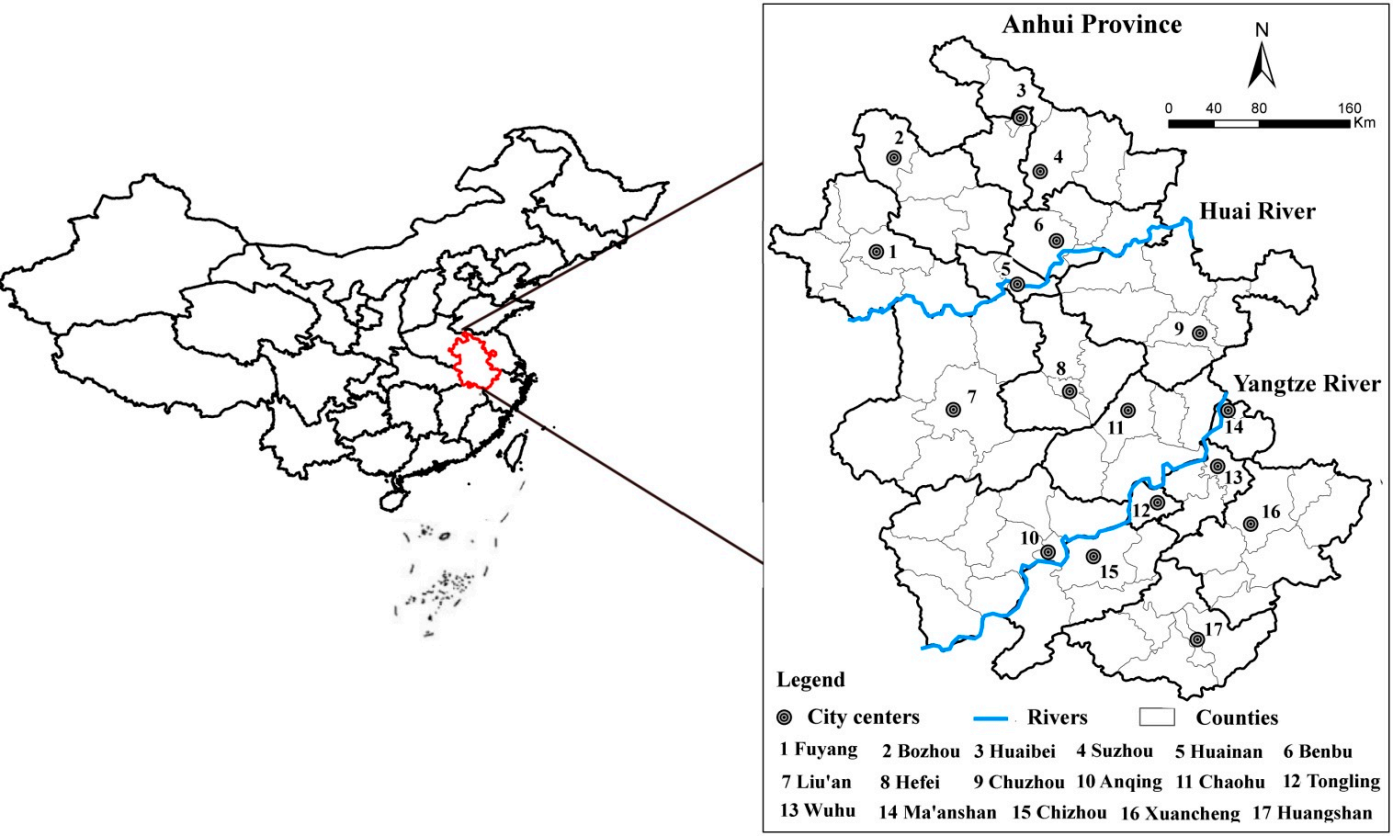
Location of the study area and its sub-areas.

Anhui Province, an inland province in Southeast China (114.54°–119.37° E, 29.41°–34.38° N), was selected as the case study area for the application of the relative sensitivity indicator. Anhui Province has a high incidence of many infectious diseases, and encompasses different climates because it locates across the basins of the Yangtze and Huai Rivers (Figure 3). The northern part of the province is part of the North China Plain, which is more temperate and with more clear-cut seasons. In contrast, the southern part is warmer and has more rainfall. In terms of administrative districts, Anhui Province is composed of 17 cities and 61 counties (hereafter all administration units are referred to as counties).

Both the infectious diseases data and climate data at the county level were collected. Monthly cases of thirteen infectious diseases at the county level from 2004 to 2010 in Anhui Province were collected from an Internet-based infectious disease reporting system coordinated by the Chinese Center for Disease Control and Prevention (China CDC). The system provides a standardized platform for data collection throughout the entire nation, and ensures data quality and reliability much better than before. In Anhui Province, the infectious diseases include: dysentery, hand, foot and mouth disease (HFM), hepatitis A, hemorrhagic fever, typhoid fever, malaria, meningitis, influenza, schistosomiasis, Japanese encephalitis, avian influenza, leptospirosis and cholera. Months without reported incidences of a disease were excluded. The monthly incidence of each disease was calculated by dividing monthly cases through the corresponding population for each county except for HFM. For this illness, we divided the monthly cases by the number of children younger than 9 years old (0, 9) because of the special population that suffers from this disease [22]. 

We also collected monthly data concerning climate variables for each county at a spatial resolution of 5 km. The dataset was generated based on field observation, remotely sensed data and reanalysis data from the Land Surface Model over the Chinese mainland (which dates from 1960 to 2010) [23]. In the study, three climate variables for the study periods were considered; monthly average temperature (MAT), monthly accumulated precipitation (MAP), and monthly average absolute humidity (MAAH). Moreover, county-level historical mean values (*HC_i_^mean^*) and the standard deviation (*HC_i_^std^*) of the three climate variables from 1973 to 2003 (30 years) were also calculated for identifying the exposure and non-exposure groups. Considering the statistical requirements of the linear regression method and the hypothesis test, only counties with at least 20 months of data concerning infectious disease, of which more than 5 and 7 months are in exposure and non-exposure groups, respectively, were regarded as the effective counties to be analyzed. The calculations were carried out using MATLAB 7.10.0 (R2010a).

### 3.2. Results

#### 3.2.1. Characteristics of Climate Variables in Exposure and Non-exposure Group

Table 2 shows the descriptive statistics for the differences in climate variables including temperature, precipitation and absolute humidity in exposure and non-exposure groups. 

**Table 2:** Descriptive statistics for the climate variables in the exposure and non-exposure groups in Anhui province.

Climate Variables	Groups	| *PC_i_* − *HC_i_^mean^*|			
		Average	Maximum	Minimum	Standard Deviation
Temperature (°C)	Exposure	2.11	4.57	0.75	0.76
	Non-exposure	0.53	1.63	0.0001	0.34
Precipitation (m)	Exposure	5.85	40.47	0.15	4.63
	Non-exposure	1.84	17.56	0.0008	1.96
Absolute Humidity (mg/L)	Exposure	115.24	253.05	34.65	44.69
	Non-exposure	38.30	115.15	0.002	25.35

Taking temperature as an example, the averaged difference between the monthly average temperatures and the historical monthly mean temperatures is 0.53 °C in the non-exposure group with a maximum and standard deviation of 1.63 °C and 0.34 °C, respectively. In contrast, in the exposure group, the corresponding average difference is 2.11 °C, with a maximum value and standard deviation of 4.57 °C and 0.76 °C, respectively. Generally, the average difference in the exposure group is more than 2 times over that of the non-exposure group’s. There are also similar trends for precipitation and absolute humidity in Anhui Province.

#### 3.2.2. Screening Results for Climate Change-Sensitive Disease

Among the above thirteen infectious diseases, nine (dysentery, HFM, hepatitis A, hemorrhagic fever, typhoid fever, malaria, meningitis, influenza, and schistosomiasis) were examined to investigate whether they would be affected by changes in the selected climatic variables based on the Relative Sensitivity indicator. The remaining four infectious diseases (Japanese encephalitis, avian influenza, leptospirosis and cholera) were excluded from the analysis due to their lower incidences during the 2004–2010 time period. Table 3 shows the Relative Sensitivity (RS) of the nine infectious diseases to changes in climate variables, as exposure factors in Anhui Province. Relative Sensitivity values where *RS* ≠ 0 and *p* < 0.05 represent significantly different associations between the exposed climate variable and the disease outcome in the exposure and non-exposure groups.

Considering that increasing temperature along with climate change is the main exposure climatic variable, the *RS* results suggested that six infectious diseases were affected by temperature change in more than 50% of the effective counties and cities in Anhui Province from 2004 to 2010; they are: HFM, malaria, influenza, typhoid fever, meningitis, and schistosomiasis. In contrast, dysentery, hepatitis A and hemorrhagic fever were affected in less than 50% of the effective counties and cities. Special hemorrhagic fever was affected in only 10% of the effective counties. 

**Table 3:** The screening results for climate change-sensitive disease using RS indicator in Anhui province.

Diseases	Monthly Average Temperature		Monthly Accumulated Precipitation		Monthly Average Absolute Humidity	
	Effective Counties	Counties with RS ≠ 0 ^*^ (Proportion)	Effective Counties	Counties with RS ≠ 0 ^*^ (Proportion)	Effective Counties	Counties with RS ≠ 0 ^*^ (Proportion)
Dysentery	77	35 (45%)	77	51 (66%)	77	73 (95%)
Hand, foot and mouth	76	48 (63%)	76	33 (43%)	76	72 (95%)
Hepatitis A	69	33 (48%)	68	40 (59%)	68	37 (54%)
Malaria	36	21 (59%)	36	10 (28%)	36	30 (83%)
Influenza	29	20 (69%)	29	10 (34%)	29	18 (62%)
Typhoid fever	16	10 (63%)	15	9 (60%)	16	10 (63%)
Hemorrhagic fever	10	1 (10%)	10	2 (20%)	10	6 (60%)
Meningitis	8	4(50%)	8	3 (38%)	8	7 (88%)
Schistosomiasis	9	6 (67%)	7	4 (57%)	9	5 (56%)

* *p*-value is less than 0.05.

By comparing the *RS* calculated from the disease incidence and precipitation in the exposure and non-exposure groups, the relative sensitivity indicator showed that precipitation change has direct or indirect effects on the sensitivities of dysentery, hepatitis A, typhoid fever and schistosomiasis in more than 50% of the effective counties in Anhui Province. For the diseases of HFM, malaria, influenza, meningitis, and hemorrhagic fever, most effective counties (67%–80%) had not witnessed the impact of precipitation change on disease epidemics in Anhui Province.

When the absolute humidity was taken as the exposed factor, the *RS* values showed that the change of absolute humidity produced an effect on all the nine infectious diseases, including dysentery, HFM, hepatitis A, hemorrhagic fever, typhoid fever, malaria, meningitis, influenza and schistosomiasis, in more than 50% of the effective counties in Anhui province. 95% of the effective counties experienced different sensitivities to dysentery and HFM to absolute humidity at two different climate statuses in the exposure and non-exposure groups. 

#### 3.2.3. Spatial Distribution for RS of the Climate Change-Sensitive Disease

**Figure 4:**
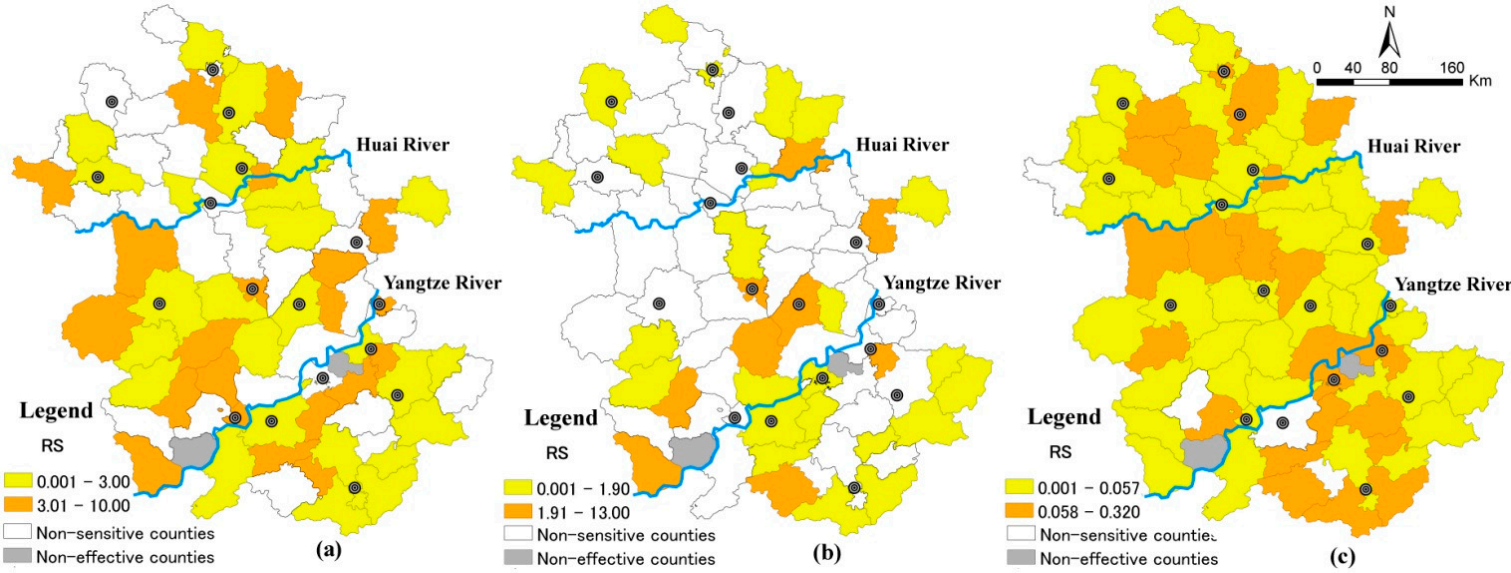
The spatial distribution of *RS* for HFM in Anhui Province: (**a**) RS to temperature; (**b**) RS to precipitation; (**c**) RS to absolute humidity.

**Figure 5:**
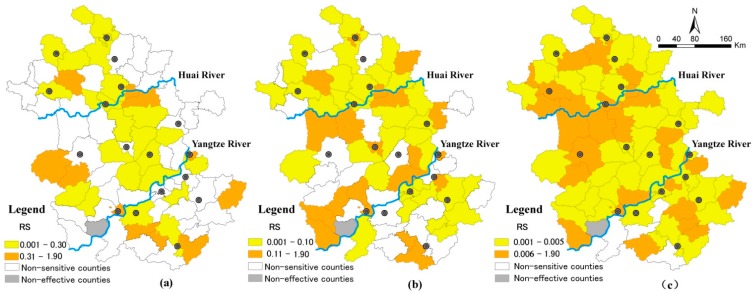
The spatial distribution of RS for dysentery in Anhui Province: (**a**) RS to temperature; (**b**) RS to precipitation; (**c**) RS to absolute humidity.

Besides finding out which diseases’ transmission would be affected by climate change, we also investigated where infectious diseases were more sensitive to climate change, in support of the prevention of these infectious diseases in local areas. Here, the top two infectious disease, *i.e.*, HFMs and dysenteries were employed to explain the spatial distribution of the relative sensitivity indicator in Figure 4 and Figure 5. 

In Anhui Province, a total of 169,287 HFM cases and 92,047 dysentery cases were reported from 2004 to 2010. In spatial distribution, the prevalence of HFM was sensitive to temperature change in 63% of the effective counties, which are scattered around the northeast, midwest and south parts of Anhui Province (Figure 4a). 

It is worthwhile to note that the relative sensitivity of HFM in eight cities among the twelve effective cities was smaller compared to that in the surrounding rural counties. There was also a similar phenomenon in the effect of absolute humidity change on HFM. The incidence of HFM in eleven cities of the sixteen effective cities were less sensitive than that in the nearby rural counties to the change of absolute humidity. It is evident that better medical conditions in cities *versus* rural counties can reduce sensitivity of climate change to some extent. 

As for dysentery, the thirty five counties where disease outbreak was sensitive to the temperature change were mainly located along a band distribution from the northwest to southeast. The counties with significantly different association between dysentery incidence and precipitation in the exposure and non-exposure groups were mainly located along the Huai River and Yangtze River. For most parts of Anhui Province, except some southwestern counties, dysentery incidence was sensitive to the change of absolute humidity. Moreover, the relative sensitivity to absolute humidity in thirteen urban cities was smaller than that in the surrounding rural counties. That is to say, rural areas may be the key areas that need more inputs in response to climate change due to the relatively underdeveloped medical conditions. 

### 3.3. Discussions

To our knowledge, this is the first study on identifying the sensitivity of the incidence of many infectious diseases to climate change in an area based on a statistical indicator. There are three main strengths of the Relative Sensitivity indicator for screening climate-change sensitive diseases. First, it could reduce the influence of seasonality and inter-annual trends in the data to some extent. It is well noticed that the original data, especially the climatic data, has notable seasonality and inter-annual trends (see Figure 2a). Thus, the existing methods (e.g., correlation analysis) always require essential data pre-processing to avoid these influences. However, when calculating Relative Sensitivity, the exposure and non-exposure groups were defined according to the difference of the climate variable between the month of interest and the historical mean value of that month, which could considerably reduce the impact of seasonality and trend. Second, Relative Sensitivity is a comprehensive indicator with a statistical basis for screening climate-change sensitive diseases, *i.e.*, the sensitivity difference between different groups (*i.e.*, exposure and non-exposure groups), is strictly examined by a well-developed hypothesis test (*F*-test). Lastly, Relative Sensitivity could be adopted for short time series datasets. As mentioned before, one of the limitations for the correlation analysis methods is that they require a relatively long and continuous time series dataset for a reliable result. Therefore, the unavailability and discontinuity of long-term epidemic data seriously limit their applications. On the contrary, Relative Sensitivity could work well, even if the time series is relatively short and discontinuous in the temporal domain. Specifically, Relative Sensitivity only needs more than five data points in each group for the linear regression model, although we acknowledge that more data points could improve the reliability of relative sensitivity to some extent.

Besides the advantages mentioned above, there are also several limitations that should be well studied in the future. One is that Relative Sensitivity is now designed for only one climate variable. In fact, changes in multiple climate variables could affect infectious diseases simultaneously. This issue should be well addressed in the future. Fortunately, Relative Sensitivity could be easily expanded to multiple climate variables, since the hypothesis test and linear regression model, as two crucial steps for Relative Sensitivity, for multiple variables have been well established. In addition, the current definition of Relative Sensitivity is the absolute value of differences between exposure and non-exposure groups, which loses the direction of changes of the infectious diseases caused by climate change. Thus, an improved version of Relative Sensitivity should be developed by considering both the magnitude and direction of relative sensitivity caused by climate change. Additionally, the Relative Sensitivity does not consider the temporal autocorrelation in incidence data. There are several reasons that might lead to temporal autocorrelation in incidence data. One reason is that there might be a time gap between getting infected and showing symptoms, which varies for different diseases. For diseases with long time gaps, the disease incidence for one month might be the result from infections during previous months. However, temporal autocorrelation caused by this could be partially avoided by using prior knowledge about specific diseases in epidemiology, such as applying lag days between getting infected and showing symptoms (or reporting incidence) to artificially match incidence data with climate variables of preceding months. Another reason for temporal autocorrelation is that high incidence in one month might increase transmission potential in subsequent months. This factor is difficult to address by statistical methods, since it is impossible to quantify this transmission potential using monthly aggregated incidence data. In order to quantify this influence, a dynamic model characterizing transmission process might be helpful. In further studies, combining transmission dynamic modeling and statistical approaches might be a potential way to address this issue.

Other studies have also assessed the influence of climate change on the incidence of different infectious diseases adopting different methods. Consistent with previous studies, empirical data analysis also showed that one or more climate variables could lead to more disease cases in some places [24,25]. For example, temperature was significantly correlated with the incidence of HFM [26,27,28], malaria [29,30], influenza [31,32], typhoid fever [33,34], meningitis [35], and schistosomiasis [36]. Precipitation change has been shown different effects on sensitivity of dysentery [37,38], hepatitis A [39], typhoid fever [33,39], and schistosomiasis [36,40]. Researchers are also interested in the relationship between humidity and infectious disease outbreaks, but most of the time relative humidity is employed [41], such as the effect of relative humidity on dysentery [38], HFM [25], malaria [29], typhoid fever [42], and so on. Relative humidity measures the ratio of water vapor content in the air to the saturating level, which varies with temperature. By contrast, absolute humidity measures the ratio of actual amount of water present in the air, without considering the temperature [43]. Shaman found that absolute humidity constrains both transmission efficiency and virus survival much more significantly than relative humidity in influenza epidemics [43]. Besides the emerging hypothesis that absolute humidity is an important determinant of observed influenza outcomes [31,43,44,45], this study provides novel ecologic evidence that absolute humidity has also a critical influence on many observed infectious diseases in Anhui Province, including dysentery, HFM, hepatitis A, hemorrhagic fever, typhoid fever, malaria, meningitis and schistosomiasis.

Although the impacts of climate change on infectious diseases are detected worldwide, the forms and degree of impacts differ greatly. Because of the complex interactions process between climate variables and infectious diseases’ components (pathogen, host and transmission environment) for the specific diseases [4], the impacts are different at different geographic scales for different infectious diseases, and sometimes controversial [46,47]. Moreover, the incidence of infectious disease is tied to many other factors besides climate, such as the location of the respective countries and socio-economical situations, pollution, and healthcare conditions [46]. In this study, we also found that the Relative Sensitivity of infectious diseases to climate change in Anhui province is different in space. For example, dysentery and HFM are more sensitive to climate change in rural areas than that in urban areas. Taking HFM for example, it is a childhood disease caused by human enteroviruses, particularly coxsackie viruses and human enterovirus 71 (HEV71) [26]. Different thermal effects on HEV71 rely on the local sensitivity of temperature and humidity, or resistant serotype [48,49]. Warmer and moister environment was helpful to prolong the viability of HEV71 and the transmission of HFM [26]. At the same time, lower temperature could decrease the gathering activity and cross infection between children, thus interrupting the disease transmission. Many researchers measured the correlation between climate variables and HFM and reported a 1.4% to 36% increase of disease incidence per 1 °C increment of temperature [26,50,51,52] and a 0.5% to 4.7% increase for every 1% increase in relative humidity [50,51]. On the other hand, some non-climatic determinants could impact the transmission of HFM, such as the child population density, good hygiene practices, and disease surveillance systems [26,46]. Importantly, there was an obvious difference of these non-climatic determinants in Anhui province, for example, the expenditure on food and health care was much higher in urban areas than that in rural areas [53].

It is important to answer why certain diseases are more sensitive than others to specific climate variables. However this requires deeper understanding of the transmission mechanism of infectious disease and more data on the pathogens and host. It is beyond the scope of this study and should be addressed in the future research.

## 4. Conclusions

This study screened different kinds of climate change-sensitive disease by defining a new statistical indicator, "Relative Sensitivity". A statistical significance test for this indicator helps to identify a significant change in the relationship between disease and climate variables. The application results in Anhui province found a significant sensitivity of the changes of climatic variables, such as temperature, precipitation and absolute humidity, to the epidemiology of many diseases. In detail, from 2004 to 2010, in more than 50% of the effective counties of Anhui Province, dysentery and hepatitis A were both sensitive to changes in precipitation and absolute humidity; HFM, malaria, influenza and meningitis were all sensitive to changes in temperature and absolute humidity; typhoid fever and schistosomiasis were both sensitive to changes in temperature, precipitation and absolute humidity; hemorrhagic fever was sensitive to a change in absolute humidity. As novel evidence, this research shows that absolute humidity has a critical influence on many observed infectious diseases in Anhui province, including dysentery, HFM, hepatitis A, hemorrhagic fever, typhoid fever, malaria, meningitis, influenza and schistosomiasis. Moreover, some infectious diseases are more sensitive to climate change in rural areas than that in urban areas. 
